# Epidemiological and clinical characteristics of hereditary angioedema in the Baltic states

**DOI:** 10.3389/fimmu.2026.1696479

**Published:** 2026-02-25

**Authors:** Natalja Kurjane, Adine Kanepa, Signe Purina, Lasma Lapina, Krista Ress, Marget Savisaar, Edita Gasiuniene, Ieva Bajoriuniene, Kotryna Linauskiene, Anzelika Chomiciene, Brigita Gradauskiene, Laura Malinauskiene

**Affiliations:** 1 Department of Biology and Microbiology, Institute of Oncology and Molecular Genetics, Riga Stradiņš University, Riga, Latvia; 2 Center for Clinical Immunology and Allergology, Pauls Stradiņš Clinical University Hospital, Riga, Latvia; 3 Allergic Diseases Diagnosis and Treatment Centre, Riga, Latvia; 4 Centre of Allergology and Immunology, East Tallinn Central Hospital, Tallinn, Estonia; 5 Lung Clinic, Tartu University Hospital, Tartu, Estonia; 6 Department of Immunology and Allergology, Lithuanian University of Health Sciences, Kaunas, Lithuania; 7 Faculty of Medicine, Institute of Clinical Medicine, Clinic of Chest Diseases, Immunology and Allergology, Vilnius University, Vilnius, Lithuania

**Keywords:** hereditary angioedema, epidemiology, prevalence, Estonia, Latvia, Lithuania

## Abstract

**Background:**

Hereditary angioedema (HAE) is a rare, potentially life-threatening disorder characterised by recurrent episodes of localised oedema caused by bradykinin overproduction. Accurate epidemiological data are essential for optimising diagnosis and treatment, particularly in underrepresented regions such as the Baltic states. This study aimed to examine the prevalence, clinical characteristics, genetic variants, and treatment accessibility for patients with HAE in the Baltic states of Estonia, Latvia, and Lithuania.

**Methods:**

This retrospective study included HAE patients diagnosed according to the WAO/EAACI 2021 criteria between 2004 and 2024. Demographic, clinical, and genetic data were collected and evaluated. Descriptive statistical analysis was performed using Jamovi (version 2.3).

**Results:**

A total of 78 patients were identified in Estonia (n=30), Latvia (n=12) and Lithuania (n=36) from 2004 till 2024. In Lithuania, 7 patients had died and 3 were lost to follow-up, resulting in 26 patients remaining under active observation. While the total number of HAE cases identified across the three countries was reported, detailed clinical data and analyses were limited to the 68 patients who were alive and actively followed at the time of data collection. Estonia exhibited the highest point prevalence (2.19 per 100, 000), while Latvia had the lowest (0.65). The median diagnostic delay was longest in Latvia (24 years) and shortest in Estonia (9.5 years). *SERPING1* gene pathogenic variants predominated. Estonia had the broadest availability of treatments, whereas Latvia had restricted access to modern therapies.

**Conclusion:**

Considerable variation exists in HAE prevalence, diagnosis, and treatment across the Baltic states. Estonia exemplifies best practices, while Latvia remains underserved. Regional collaboration and standardised care protocols are urgently needed.

## 1 Introduction

Hereditary angioedema (HAE) is a rare, monogenic disorder characterised by spontaneous or provoked angioedema attacks that can affect various body parts, including the face, upper airways, limbs, genitals, and gastrointestinal tract. HAE poses a life-threatening risk, particularly when oedema occurs in the upper airways, potentially leading to asphyxiation. In most cases, the primary pathological mechanism involves bradykinin-induced blood vessel leakage, resulting in localised swelling and angioedema in various parts of the body (1).

HAE is categorised into two primary types: HAE due to C1-inhibitor deficiency (HAE-C1INH; types I and II) and hereditary angioedema with normal C1-inhibitor (HAE-nC1INH). Each type is distinguished by specific genetic variations and clinical presentations (2, 3). HAE-C1INH-Type 1 is the most prevalent form, accounting for approximately 80–85% of the patients. It is characterised by decreased levels of both the functional and antigenic C1 inhibitor (C1-INH) in the blood, typically due to pathogenic variants in the SERPING1 gene. HAE- C1INH-Type 2, which accounts for approximately 10–15% of patients, is characterised by the production of dysfunctional C1-INH despite normal serum levels, often resulting from protein-altering variants (e.g., missense) in the C1-INH protein’s active centre (3–7).

In recent years, substantial research efforts have been dedicated to delineating the genetic foundations and pathophysiological aspects of HAE-nC1INH. Despite these efforts, the aetiology in most cases remains elusive. Several genes, including *coagulation factor XII (F12), plasminogen (PLG), angiopoietin 1 (ANGPT1), kininogen 1 (KNG1), myoferlin (MYOF), heparan sulphate-glucosamine 3-sulfotransferase 6 (HS3ST6), carboxypeptidase N (CPN1) and disabled homolog 2 interacting protein (DAB2IP)* have been implicated in the condition (8–16). However, except for *F12* and *PLG*, the findings are typically confined to individual families and have not been replicated in unrelated cohorts.

HAE is often poorly recognised due to its nonspecific symptoms, leading to delays in appropriate therapy and an increased risk of morbidity and mortality in patients. However, certain characteristic clinical features — including recurrent episodes of non-urticarial angioedema involving the skin, gastrointestinal tract, or upper airway, recurrent abdominal pain often accompanied by nausea and vomiting, absence of pruritus or urticaria, poor or absent response to antihistamines, corticosteroids, or epinephrine, and a positive family history of angioedema, can facilitate an earlier and more accurate diagnosis of HAE.

This study aimed to describe the prevalence and clinical diversity of HAE in the Baltic states, specifically in Estonia, Latvia, and Lithuania.

## 2 Methods

A retrospective descriptive study was conducted across the three Baltic states: Estonia, Latvia, and Lithuania. All patients from these countries diagnosed with HAE according to the WAO/EAACI 2021 criteria between 2004 and 2024 were included in the study. The diagnosis of HAE was recorded in the official health statistics of all three Baltic countries using the International Classification of Diseases, 10th Revision (ICD-10).

All laboratory analyses were performed in reference or university hospital laboratories across Lithuania, Latvia, and Estonia. Genetic testing was also performed in laboratories across the Baltic countries, including the Genetics and Molecular Medicine Department at the Hospital of Lithuanian University of Health Sciences Kauno klinikos, and Vilnius University Hospital Santaros Klinikos; the Scientific Laboratory of Molecular Genetics at Riga Stradiņš University in Latvia; and the Department of Laboratory Genetics at Tartu University Hospital in Estonia, as well as by Asper Biogene LLC. The diagnostic work-up included evaluation of clinical history and assessment of serum C4 levels, quantitative and functional measurements of C1 esterase inhibitor (C1-INH), and C1q levels to exclude acquired forms of angioedema. Genetic testing was performed to identify pathogenic variants in *SERPING1, F12, PLG, ANGPT1, KNG1, MYOF*, and *HS3ST6* (although not in all cases).

### 2.1 Collection of demographic data from the general population

Data on the demographic characteristics of the general population were collected from the Central Statistical Bureau of Latvia, the Lithuanian Official Statistics Portal, and Statistics Estonia (December 2024). The point prevalence was expressed as living patients per 100, 000 inhabitants at a particular point in time (December 2024).

### 2.2 Collection of clinical data

Data from medical records, including age, symptom onset, symptom severity, and diagnostic delay (see **Table 1**), were collected and evaluated. Laboratory test results (complement components and genetic findings) were used to diagnose HAE according to the global WAO/EAACI 2021 criteria. Patient consent was not required for this study due to its retrospective design.

**Table 1:** Characteristics of HAE in the Baltic states from 2004 till 2024.

	Estonia	Latvia	Lithuania
Number of HAE patients (males and females)	30	12	36* (active 26)
Number of children	4	1	3
Median age, years	47 (range: 5–95; IQR: 29.3)	40 (range: 1–65; IQR: 36.5–58.9)	49 (range: 22−75; IQR: 20.5)
Females (including children)	14 (47%)	8 (67%)	18 (69%)
Males (including children)	16 (53%)	4 (33%)	8 (31%)
HAE-C1INH-Type 1	26 (86%)	10 (83%)	24 (92%)
HAE-C1INH-Type 2	2 (7%)	1 (8, 5%)	0 (0%)
HAE-nC1INH	2 (7%)	1 (8, 5%)	2 (8%)
Family history: yes	26 (86%)	8 (66%)	16 (61, 5%)
The median age at onset of symptoms, years	15 (IQR**: 18.5)	15 (IQR: 6.25–16.75)	23 (IQR:22.0)
The median age at diagnosis, years	35 (IQR: 24.3)	39 (IQR: 25.5–49.8)	41 (IQR:28.0)
The median time from symptoms to diagnosis, years	9.5 (IQR:14.8)	24 (IQR: 14.7–34.5)	16 (IQR:11.5)

*In Kaunas Centre: 5 patients deceased, 3 lost to follow-up; in the Vilnius Centre: 2 patients deceased.

**IQR, Interquartile Range.

### 2.3 Ethics

The study was approved by the Ethical Committees in all three Baltic countries. The Latvian data analysis was approved by the Latvian Central Medical Ethics Committee (No. 01-29.1/2878, dated 03/06/2020) and conforms to the principles of the Declaration of Helsinki. The Lithuanian data analysis from the National Registry was conducted with informed consent (No. BE-2-12, Kaunas Regional Biomedical Research Committee) and approved by the Vilnius Regional Bioethics Committee (approval No. 158200-16-847-355). The Estonian data analysis was approved by the NIH Human Research Ethics Committee (approval No. 1313).

### 2.4 Statistical analysis

The demographic data of the patients were analysed using descriptive statistics. Statistical analysis was performed using Jamovi (version 2.3). The point prevalence was calculated per 100, 000 inhabitants at a particular point in time (March 2024).

## 3 Results

A total of 78 patients were diagnosed with HAE between 2004 and 2024 across the three Baltic states: 30 in Estonia, 12 in Latvia and 36 in Lithuania. Of the 36 Lithuanian patients, 10 were not included in the analysis due to 7 deaths (5 in Kaunas and 2 in Vilnius) and 3 patients lost to follow-up, as data on presenting symptoms and disease course were unavailable. Thus, only 26 Lithuanian patients were included in the current analysis. While a total of 78 HAE cases were identified across the three countries, detailed clinical data and analyses were limited to the 68 patients who were alive and under active follow-up at the time of data collection.

Of the deceased HAE patients from Lithuania, three deaths were directly related to HAE, two were unrelated to the disease, and the cause of death remained unknown in the remaining cases. Five deaths were documented in Kaunas (Lithuania): one patient died due to an acute cardiac event unrelated to HAE; one death was attributed to probable laryngeal angioedema; one resulted from an abdominal HAE episode complicated by intestinal necrosis; and the cause of death in two cases remained undetermined. In Vilnius (Lithuania), one patient died following an HAE attack, while the other died from pulmonary thrombosis, most likely secondary to adrenal gland carcinoma.

The point prevalence was highest in Estonia (2.19 per 100, 000), followed by Lithuania (0.9 per 100, 000) and Latvia (0.65 per 100, 000).

The median age at diagnosis varied: 47 in Estonia, 40 years in Latvia and 49 in Lithuania. Median diagnostic delays were significant, particularly in Latvia, where the average time from symptom onset to diagnosis was 24 years, compared to 16.3 years in Lithuania and 9.5 years in Estonia. This suggests systemic differences in clinical recognition and referral patterns.

Most patients were diagnosed with HAE-C1INH-Type 1: 26 (86%) in Estonia, 10 (83%) in Latvia and 24 (92%) in Lithuania. HAE-C1INH-Type 2 was less common, being identified in two patients in Estonia, one in Latvia, and none in Lithuania. And only 5 patients (1 in Latvia, 2 in Lithuania, 2 in Estonia) had HAE with normal C1-INH (HAE-nC1INH). A positive family history was reported in 66% of Latvian patients, 61, 5% of Lithuanian patients, and 86% of Estonian patients, indicating effective familial screening in Estonia.

Attack frequency and severity varied. Latvia reported the highest proportion of patients experiencing more than 24 attacks per year, while Estonia had the highest number of asymptomatic patients at diagnosis. The anatomical sites most affected included extremities and abdomen. Facial and laryngeal involvement was more frequent in Latvia and Lithuania. The common triggers included stress, infections, and mechanical trauma (**Table 2**).

**Table 2:** Clinical features of the HAE patients (excluding children).

	Estonia	Latvia	Lithuania
Average annual attack frequency at diagnosis of HAE			
Asymptomatic	5	1	4
1-5	7	2	6
6-11	5	3	6
12-24	7	0	5
>24	2	5	2
Intensity of attacks in the previous 12 months			
Mild (1–3 attacks per year)	7	2	7
Moderate (4–11 attacks per year)	7	3	9
Severe (≥12 attacks per year)	2	5	7
Asymptomatic	10	1	0
Affected area			
Face (lips, cheeks, orbital)	5	9	1
Tongue	1	7	12
Larynx/throat	5	7	5
Abdomen	18	7	7
Extremities	22	10	18
Urogenital	5	3	2
Prodrome			
Tiredness	1	8	11
Paraesthesia/pain	4	8	2
Nausea	0	3	2
Erythema marginatum	5	2	1
Trigger factors			
Stress	13	5	15
Infection	9	1	14
Trauma	8	3	14
Surgical/dental manipulations	2	2	11
Mechanical pressure	12	3	11
Menstruation	3	1	1
ACE inhibitors	1	0	3
Oral oestrogen-containing contraceptives	0	1	2

Genetic analysis revealed a heterogeneous spectrum of *SERPING1* pathogenic variants in all countries, including missense, nonsense, splice-site, and frameshift variants. Latvia and Lithuania share a recurrent frameshift variant (c.1312del p.(Val438PhefsTer12)), supporting a possible founder effect. Estonia’s cohort demonstrates greater genetic diversity (**Table 3**).

**Table 3:** Genetic variants identified in HAE patients from Lithuania, Estonia, and Latvia.

Gene	Variant (cDNA / protein)	Effect	Classification	Country
SERPING1	c.816_818del p.Asn272del	In-frame deletion	Pathogenic	LT
SERPING1	c.1351G>T p.Gly451*	Nonsense	Pathogenic	LT
SERPING1	c.47C>G p.Thr158Arg	Missense	Likely pathogenic	LT
SERPING1	c.536C>T p.Ala179Val	Missense	VUS	LT
SERPING1	c.1189A>C p.Thr397Pro	Missense	VUS / likely benign	LT
MYOF	c.1312dup p.Asn438Lysfs*4	Frameshift	VUS	LT
SERPING1	c.76delG p.Ala26Leufs*53	Frameshift	Pathogenic	EE
SERPING1	c.1109T>A p.Met370Lys	Missense	Likely pathogenic	EE
SERPING1	c.1029+1G>A (IVS6 + 1G>A)	Canonical splice donor	Pathogenic	EE
SERPING1	c.1480C>G p.Arg494Gly	Missense	VUS	EE
SERPING1	c.1396C>T p.Arg466Cys	Missense	Pathogenic	EE
SERPING1	c.1312del p.Val438Phefs*12	Frameshift	Likely pathogenic	LV
SERPING1	c.1249+4A>G	Splice region	Likely pathogenic	LV
SERPING1	c.1396C>T p.Arg466Cys	Missense	Pathogenic	LV
SERPING1	c.1195C>T p.Pro399Ser	Missense	Pathogenic	LV
SERPING1	chr11:g.57600729_57603011del	Large gene deletion	Pathogenic	LV
SERPING1	c.550G>A p.Gly184Arg	Missense (possible splicing effect)	Pathogenic	LV
SERPING1	c.1136T>C p.Phe379Ser	Missense	VUS	LV
PLG	c.988A>G p.Lys330Glu (K330E)	Missense	Pathogenic	LV

VUS, variants of uncertain significance; LT, Lithuania; EE, Estonia; LV, Latvia; MYOF, myoferlin; PLG, plasminogen.

Access to treatment varied significantly. Icatibant and fresh frozen plasma were universally available. However, Latvia lacked access to plasma-derived and recombinant C1-INH products as well as newer prophylactic agents such as lanadelumab and berotralstat. Estonia provided the most comprehensive therapeutic arsenal, aligning closely with international treatment guidelines.

## 4 Discussion

This study provides the first comprehensive, multicentre, long-term evaluation of hereditary angioedema (HAE) across Latvia, Lithuania, and Estonia, highlighting both shared and country-specific patterns in epidemiology, clinical management, and genetic characteristics. Until 2025, the Baltic region lacked a functional network of specialised HAE centres, care was delivered primarily within primary immunodeficiency (PID) units or general Allergy and Immunology departments. In Latvia, the ACARE centre was established in October 2025, marking the first formal step toward structured, specialised HAE care.

In our study, Latvia demonstrated the lowest point prevalence at 0.65 per 100, 000 inhabitants, with only 12 identified patients, primarily HAE-C1INH types 1 or 2 and a single HAE-nC1INH case (17, 18). Lithuania reported a prevalence of 0.9 per 100, 000, with 26 active cases from two current centres. Estonia had the highest prevalence at 2.19 per 100, 000, identifying 30 patients (26 adults, 4 children). HAE prevalence in the Baltic countries is broadly consistent with estimates reported across Europe, where rates range from 1.07–1.09 per 100, 000 in Greece and Spain to 2.4–2.6 per 100, 000 in Slovakia and Finland (25–28). A meta-analysis reported a pooled global prevalence of 1.22 per 100, 000 individuals (31), which aligns with the estimates observed in Lithuania and Estonia. In contrast, the prevalence in Latvia appears lower than expected, with Estonia approaching the higher European benchmarks while Latvia remains below them.

Diagnostic delays varied substantially— 24 years in Latvia, 16.3 years in Lithuania, and 9.5 years in Estonia, reflecting differences in disease awareness, the effectiveness of family screening, and healthcare infrastructure. Such delays pose an additional challenge and are not unique to the Baltic region. Several European studies have reported similarly prolonged diagnostic intervals, ranging from 8.5 to 15.0 years among newly diagnosed patients without a family history of HAE (29, 30). Recent improvements in Latvia’s diagnostic capacity, fully implemented within the past two years, help clarify the extent of earlier underdiagnosis. Mortality data, particularly from Lithuania, highlight the critical importance to timely diagnosis and effective therapy to prevent life-threatening complications such as laryngeal angioedema.

A positive family history was documented in 86% of Estonian, 66% of Latvian, and 61.5% of Lithuanian patients. Estonia’s high familial clustering and successful cascade screening highlight effective family-based case identification. Lithuania had a notably higher proportion of patients without a known family history (38, 5%), possibly due to *de novo* mutations or incomplete familial documentation. In all Baltic countries, screening of all first-degree relatives is recommended for newly diagnosed individuals in accordance with international guidelines. However, due to data protection regulations and ethical considerations, clinics cannot directly contact family members. Participation in screening relies on the consent and initiative of the index patient, consequently, not all patients inform or facilitate evaluation of their relatives, which may be influenced by personal, logistical, or psychological factors. Although family screening is actively encouraged and offered to all relatives, some individuals decline testing if asymptomatic or prefer not to know their genetic status, as observed in the Vilnius (Lithuania) cohort.

Median age at diagnosis was comparable—40 years in Latvia, 49 in Lithuania, and 47 in Estonia—with overlapping distributions. HAE-C1INH Type 1 dominated in all countries (83–92%), while Types 2 and nC1INH were rare. Median symptom onset ranged from 15 to 23 years. Estonia had more asymptomatic cases and a wider phenotypic range. Latvia recorded the highest proportion of frequent attacks (>24 annually), suggesting under-treatment or delayed diagnosis. Anatomical attack localization varied: Latvia and Lithuania reported more laryngeal, tongue and facial involvement critical due to the risk of airway obstruction, whereas Estonia had more abdominal episodes. Prodromal symptoms and common triggers such as stress, infection, and mechanical trauma varied, with Lithuania reporting more surgical and traumatic precipitating factors.

Treatment access revealed notable disparities across the region (**Table 4**). In Lithuania, all patients receive on-demand C1-INH, though supply rationing occurs due to reimbursement limits. Estonia offers the most comprehensive access, including modern long-term prophylactics (LTP) such as lanadelumab, berotralstat, and pdC1-INH, reflecting alignment with current international standards (19). Lithuania provides lanadelumab for LTP, with berotralstat becoming available only in 2025. Latvia continues to rely primarily on attenuated androgens and tranexamic acid, with no access to targeted biologics or newer C1-INH therapies. Icatibant is universally available across the Baltics as first-line acute therapy, while pdC1-INH is accessible in Lithuania and Estonia but not Latvia. Recombinant C1-INH is unavailable in all three countries. Fresh frozen plasma is still used as an emergency fallback option. A recent international review of data from 28 Asia-Pacific countries further underscored these global disparities. Although guideline-recommended modern therapies have significantly reduced mortality and improved quality of life in high-income countries, access to these treatments remains very limited in many low- and middle-income settings. The authors emphasized the importance of pragmatic solutions-such as optimizes use of second-line agents, strengthening patient organizations, and advocacy-to support more equitable access to effective HAE care worldwide (32).

**Table 4:** Possibilities for HAE treatment.

	Estonia	Latvia	Lithuania
Acute treatment for HAE attacks			
Icatibant	Available	Available	Available
Recombinant C1-inhibitor (conestat alfa)	–	–	–
Plasma-derived C1-inhibitor	Available	–	Available
Fresh frozen plasma	Available	Available	Available
Long-term prophylactic treatment			
Plasma-derived C1-inhibitor	Available	–	Available
Lanadelumab	Available	–	Available
Berotralstat	Available	–	Not available
Attenuated androgens	Not used	Available	Available
Tranexamic acid	Not used	Available	Available

The proportion of patients who underwent molecular diagnostic testing varied between the Baltic countries. In Latvia, for example, 100% of patients received molecular testing. By contrast, in Estonia, 90% of patients were tested, while in Lithuania, only 69% of active patients underwent testing, with many declining genetic analysis. Many publications report that loss-of-function variants in *SERPING1* are prevalent in HAE (20, 21). Results of genetic studies in three Baltic countries reflect global trends with several truncating variants (such as c.76delG, c.1312del, and a whole-gene deletion) and splice-site changes (like c.1029 + 1G>A and c.1249 + 4A>G) that are classified as pathogenic or likely pathogenic (22). Our data identified several recurrent missense variants in key functional regions of the protein, including p.Arg466Cys, p.Gly184Arg, and changes near residues 399–438. These variants have been linked to HAE-C1-INH in multiple populations and reported in variant-update publications and national cohort studies (12, 17, 18, 20, 23). In addition, the Latvian cohort contained a novel frameshift variant (c.1312del, p.Val438PhefsTer12), beginning at residue 438 and resulting in premature truncation of the protein. The identification of two variants, *PLG* p.Lys330Glu and *MYOF* p.Asn438Lysfs*4, contributes to the growing literature on HAE with normal C1-INH (8, 24). In our dataset, the Estonian cohort showed the greatest diversity of *SERPING1* variants compared to the Lithuanian and Latvian patient groups.

This analysis reveals significant intercountry differences in prevalence, disease recognition, and therapeutic access in Baltic countries. Latvia’s small patient cohort combined with prolonged diagnostic delays indicates gaps in disease awareness and limited uptake of long-term prophylactic treatment despite available appropriate diagnostic tools. Lithuania demonstrates intermediate progress in diagnosis and treatment, albeit with challenges in follow-up and potential underreporting in some regions. Estonia exemplifies robust disease control through timely diagnosis, extensive family screening, and access to advanced therapies, resulting in a higher proportion of well-controlled or asymptomatic patients. Complementary findings from another APAC study further illustrate the transformative role of patient advocacy groups (PAGs). Despite the region comprising half of the world’s population, reported HAE prevalence remains far below global estimates, indicating substantial underdiagnosis. The establishment of PAGs was associated with increases in reported prevalence, expanded diagnostic testing, and broader availability of HAE-specific medications. Following the formation of PAGs, therapy access rose from below 50% to over 80% of surveyed countries. These results demonstrate that strong advocacy networks can effectively reduce disparities and improve care—an important consideration for regions such as the Baltic states (33).

These disparities mirror broader European patterns where access to modern HAE treatments remains inconsistent. To bridge these gaps, harmonization of diagnostic protocols, regional policy alignment, expansion of genetic testing, centralised registries, and equitable drug availability are urgently required. Such measures are pivotal to improving clinical outcomes for patients affected by this rare but potentially life-threatening disorder.

## 5 Conclusion

Hereditary angioedema remains underdiagnosed and unevenly managed in parts of the Baltic states. Estonia offers a model for effective integration of diagnostics and therapy, while Latvia illustrates the need for systemic reforms.

## Data Availability

All data generated or analysed during this study are included in this published article and its supplementary information files.
